# Overexpression of the Transcription Factor Gene *OsSTAP1* Increases Salt Tolerance in Rice

**DOI:** 10.1186/s12284-020-00405-4

**Published:** 2020-07-23

**Authors:** Yinxiao Wang, Juan Wang, Xiuqin Zhao, Sheng Yang, Liyu Huang, Fengping Du, Zhikang Li, Xiangqiang Zhao, Binying Fu, Wensheng Wang

**Affiliations:** 1 Institute of Crop Sciences/National Key Facility for Crop Gene Resources and Genetic Improvement, Chinese Academy of Agricultural Sciences, Beijing, China; 2 School of Life Sciences, Nantong University, Nantong, China; 3 School of Agriculture, Yunnan University, Kunming, China; 4 Anhui Agricultural University, Hefei, China

**Keywords:** Rice, Salinity, *OsSTAP1*, Transcription factor, ABA

## Abstract

**Background:**

High soil salinity can cause significant losses in rice productivity worldwide, mainly because salt inhibits plant growth and reduces grain yield. To cope with environmental changes, plants have evolved several adaptive mechanisms that involve the regulation of many stress-responsive genes.

**Results:**

In this study, we identified *OsSTAP1*, which encodes an AP2/ERF-type transcription factor, was rapidly induced by ABA, ACC, salt, cold, and PEG treatments. OsSTAP1 is localized to the nucleus and acts as a transcriptional activator in plant cells. Compared with wild type, transgenic lines overexpressing *OsSTAP1* exhibited increased tolerance to salt stress with higher SOD, POD, and CAT activities, and lower Na^+^/K^+^ ratios in the shoots. In addition, many other stress-responsive genes, including other ERF- and peroxidase-encoding genes, were upregulated in the *OsSTAP1*-overexpression lines.

**Conclusion:**

This study suggests that OsSTAP1 functions as an AP2/ERF transcriptional activator, and plays a positive role in salt tolerance by decreasing the Na^+^/K^+^ ratio and maintaining cellular redox homeostasis.

## Background

Rice (*Oryza sativa* L.) is an important cereal crop that helps to ensure food security in China. Rice plants are salt sensitive, and suffer from salt stress when the concentration of soluble salt in the soil reaches 0.3% (Barrett 2005). There are approximately 954 million hectares of saline alkaline land in the world, of which ~ 99 million hectares are in China (Alexandrov et al. 2015). With the continuous changes in global climate and the decrease in the amount of cultivated land area in China, salinization has become the main factor that constrains both grain yield in rice and further increases in the planting area devoted to rice. Therefore, it is urgent that plant breeders develop new varieties with a high level of tolerance to salt stress.

Salt tolerance is a complex trait in plants. The physiological and biochemical changes in response to salt stress are ultimately determined by related genes, which participate in various molecular regulatory pathways and form a complex genetic regulatory network (Atkinson and Urwin 2012). All protein-coding genes involved in response to salt stress can be divided into two categories - functional genes and regulatory genes. Of the regulatory genes, several families of transcription factors (TFs), such as AP2/ERF (APETALA2/Ethylene responsive factor), bHLH (base helix-loop-helix transcription factors), NAC (NAM、ATAF1/2、CUC2 transcription factors), MYB (v-myb avian myeloblastosis viral oncogene homolog transcription factors), and bZIP (basic leucine zipper transcription factors), were found to perform pivotal functions in response to salt stress by regulating large numbers of downstream genes and pathways (Singh et al. 2002). AP2/ERF-type transcription factors are a large family with ~ 163 members in rice that play important roles in plant growth and development and also in the responses to environmental stimuli. AP2/EREBP (APETALA2/ethylene-responsive element binding protein) TFs are characterized by the presence of the AP2 DNA-binding domain of ~ 60 amino acids, and divided into the four AP2, DREB, ERF, and RAV subfamilies based on the presence of one or two AP2 DNA-binding domains (Feng et al. 2005; Okamuro et al. 1997). Most AP2/ERF-type TFs regulate the expression of different downstream genes and mediate various biological processes by binding to different cis elements in the gene promoters, including ethylene-responsive elements (ERE), drought response elements (DRE), C-repeat (CRT), and GCC box cis elements (Sharoni et al. 2011). In addition, it has been shown that ERFs can also bind to the Hypoxia-Responsive Promoter Element (HRPE: AAACCA(G/C)(G/C)(G/C)GC), ATCTA motif, and Coupling Element 1 (CE1: TGCCACCG) (Welsch et al. 2007; Gasch et al. 2016).

ERF genes can also function in abiotic and/or biotic stress response pathways. Overexpression of rice AP2/ERF-type TF genes can significantly enhance drought, salt, and cold tolerance in *Arabidopsis* (Dubouzet et al. 2003; Sun et al. 2008). TSRF1, an ERF protein from tomato, enhanced osmotic and drought tolerance in rice by up-regulating the expression of stress responsive genes (Quan et al. 2010). ERF1 and ERF6, two members of the ERF-IX group, positively regulate plant salt tolerance and confer long-term osmotic stress tolerance (Zhang et al. 2011; Dubois et al. 2013). RAP2.6 L belongs to the ERF-X subgroup and can also improve drought and salt tolerance (Yang et al. 2009; Liu et al. 2012a). It is worth noting that AP2/ERF-type TFs are involved in crosstalk between phytohormone and abiotic stress signaling pathways. *Sub1A,* a gene that encodes an AP2/ERF-type transcription factor, increases tolerance to both submergence and drought by regulating the expression of ethylene biosynthesis genes (Fukao et al. 2011). Also, *SNORKEL1* and *SNORKEL2* increase submergence tolerance via rapid and remarkable internode elongation triggered by gibberellin (Hattori et al. 2009). CYTOKININ RESPONSE FACTORS (CRFs), which are members of the ERF-VI subfamily, can be induced by CTK and positively regulate freezing and osmotic tolerance (Rashotte and Goertzen 2010). Expression of *OsERF71* can alter root architecture and confer drought tolerance mediated by ABA (abscisic acid) signaling (Li et al. 2018).

In our previous study, the AP2/ERF-type transcription factor gene *OsSTAP1* was identified in rice seedlings in response to treatment with various abiotic stresses and hormones using a microarray gene chip (Jin et al. 2009). *OsSTAP1* was located in an introgressed segment on chromosome 3, and its expression was greatly upregulated by salt stress, suggesting a potential role in salt tolerance in rice. Here, we report the functional characterization of *OsSTAP1* and provide evidence for its important role in improving salt tolerance in rice.

## Results

### Identification and Characterization of *OsSTAP1*

**Fig. 1:**
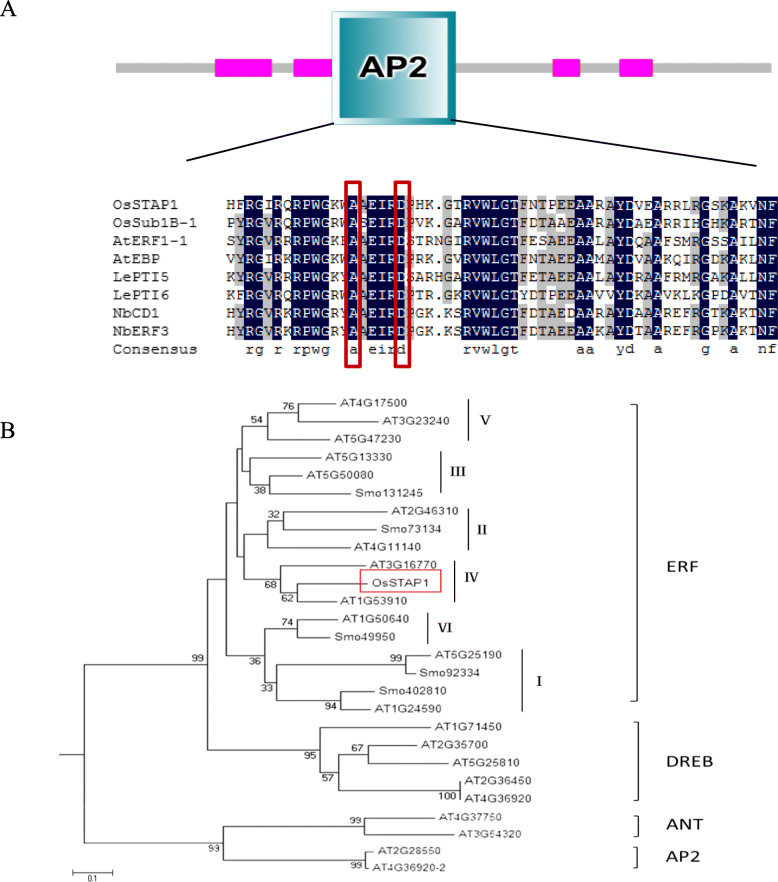
Identification and characterization of *OsSTAP1.* Structural analysis of OsSTAP1 (**a**) and a phylogenetic tree (**b**) showing the relationships among rice AP2/EREBP proteins after sequence alignment with ClustalW

Based on our previous work characterizing AP2/EREBP gene expression profiling in rice seedlings treated with PEG, low-temperature, high-salt, ABA, and GA (gibberellin) using a microarray gene chip, we identified two AP2/EREBP genes that showed the same expression pattern in response to all five stress treatments (Jin et al. 2009). One of these AP2/EREBP genes, *OsSTAP1* (LOC_Os03g08470), was found to be located on rice chromosome 3 with a coding sequence of 1005 bp. *OsSTAP1* encoded a predicted 334-amino-acid protein, and protein structure analysis showed that OsSTAP1 was an AP2/ERF-type protein with one single conserved AP2/ERF domain located between amino acids 110 and 173. Further analysis showed that the ERF/AP2 domain of OsSTAP1 contains the 124th Ala and 129th Asp residues (Fig. 1a), two key conserved amino acids in ERF-type TFs rather than 124th Val and 129th Glu in DREB-type TFs (Sakuma et al. 2002). Phylogenetic analysis based on the amino acid sequences of OsSTAP1 and other characterized plant AP2/ERF genes (Mizoi et al. 2011) showed that OsSTAP1 clustered with the ERF sub-family, particularly group 6 (Fig. 1b), and the homologous protein in *Arabidopsis thaliana* was At1g53910, an ERF-type protein. At1g53910 encoded a stress-related protein Rap2.2 and was involved in the ethylene signaling pathway (Zhao et al. 2012), which indicated that OsSTAP1 may also participate in the stress response as well as in ethylene signaling.

To investigate the regulatory roles of *OsSTAP1*, we analyzed the *cis*-acting elements in the promoter sequence (2000 bp upstream of the transcription start site) by searching the PLACE database (https://www.dna.affrc.go.jp/PLACE/?action=newplace). The results revealed that the promoter region of *OsSTAP1* contained multiple hormone response elements and stress response-related cis-acting elements including ABRE (abscisic acid response element), GARE-motif (gibberellin response element), LTR (low temperature response element), and ARFAT (auxin response factor binding site), among others (Supplementary Table S1). Such an enrichment in hormone response elements and stress response-related cis-acting elements may suggest a critical role for *OsSTAP1* in stress tolerance in rice.

### *OsSTAP1* Expression Patterns in Response to Various Abiotic Stresses and Exogenous Phytohormone Treatments

**Fig. 2:**
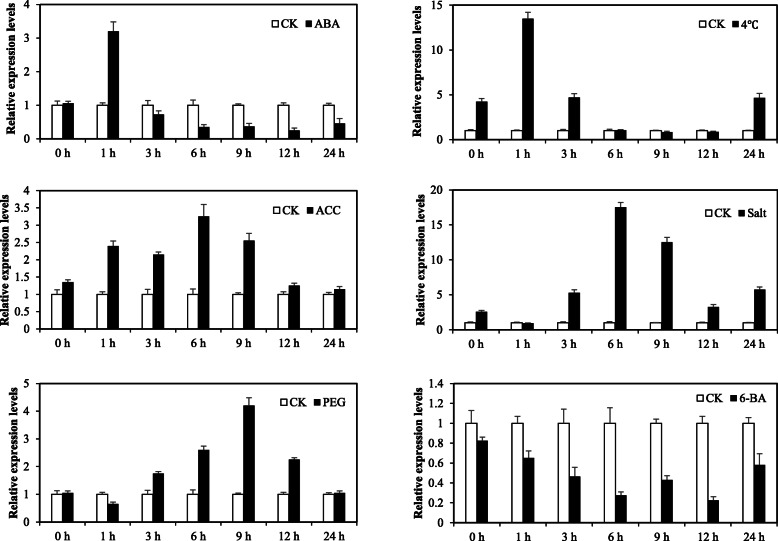
Expression profiles of *OsSTAP1* in response to various abiotic stress and phytohormones. Wild-type rice (*Oryza sativa* L. ssp. *japonica* cv. ‘Nipponbare’) plants treated with 20% PEG, 150 mM NaCl, cold (4 °C), 100 μM ABA, 100 μM ACC, and 100 μM 6-BA. Total RNA was isolated at the indicated times (hours) after each treatment. Error bars showing the SD based on three replicates. The *Actin* gene was used as the endogenous control for normalization of gene expression

It has been reported that many ERF-type TFs take part in hormonal signal transduction in response to biotic and abiotic stresses (Xie et al. 2019). To investigate whether *OsSTAP1* is involved in the rice response to various abiotic stresses and hormones, the expression patterns of *OsSTAP1* in response to treatments with PEG (Polyethylene glycol), high salt, low-temperature, ABA, 6-BA (N-(Phenylmethyl)-9H-purin-6-amine), and the ethylene precursor ACC (1-aminocyclopropane-1-carboxylate) were determined (Fig. 2). The *OsSTAP1* gene showed similar expression profiles in response to salt, ACC, cold, ABA, and PEG, first increasing and then decreasing with time, while the expression was considerably down-regulated by cytokinin (6-BA). It is worth noting that the expression of *OsSTAP1* significantly increased after treatment with ACC, ABA, or cold for 1 h. These results suggest that *OsSTAP1* could be involved in the plant early response to the hormone signaling and is associated with ethylene- and ABA-related stress responses.

### OsSTAP1 Is a Nuclear-Localized Protein and Acts as an Transcriptional Activator

**Fig. 3:**
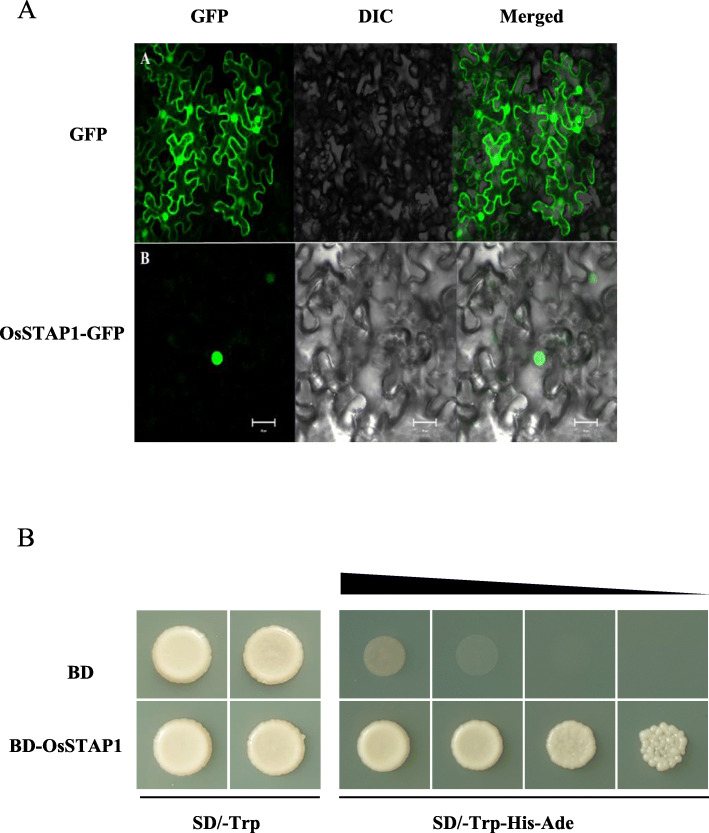
Transactivation analysis of OsSTAP1 in yeast. **a** GFP, the positive control, was distributed in the nucleus and cytoplasm, while OsSTAP1-GFP was specifically localized to the nucleus. Scale bars, 10 μm. **b** Transcriptional activity identification of OsSTAP1 in yeast with pGBKT7 as the negative control. On the medium of SD/−Trp-His-Ade, yeast suspension was diluted to 10^− 1^, 10^− 2^, 10^− 3^ times

ERF-type proteins have been shown to function as transcription factors that regulate target gene expression in the nucleus (Fujimoto et al. 2000). In order to further determine whether OsSTAP1, as an ERF-type transcription factor, is localized to the nucleus, we conducted transient expression experiments in tobacco leaf cells using an OsSTAP1-GFP (green fluorescent protein) fusion construct. Compared to the epidermal cells transformed with an empty GFP vector alone, the GFP fluorescence signal was only observed in the nucleus with the OsSTAP1-GFP fusion construct (Fig. 3a). Yeast activation tests showed that yeast cells transformed with BD-OsSTAP1 grew normally in SD/−Trp-His-Ade medium, compared to yeast with the BD control vector (Fig. 3b). These results demonstrate that OsSTAP1 is localized to the nucleus and functions as a transcriptional activator in rice.

### *OsSTAP1* Positively Regulates Salt Tolerance in Rice

**Fig. 4:**
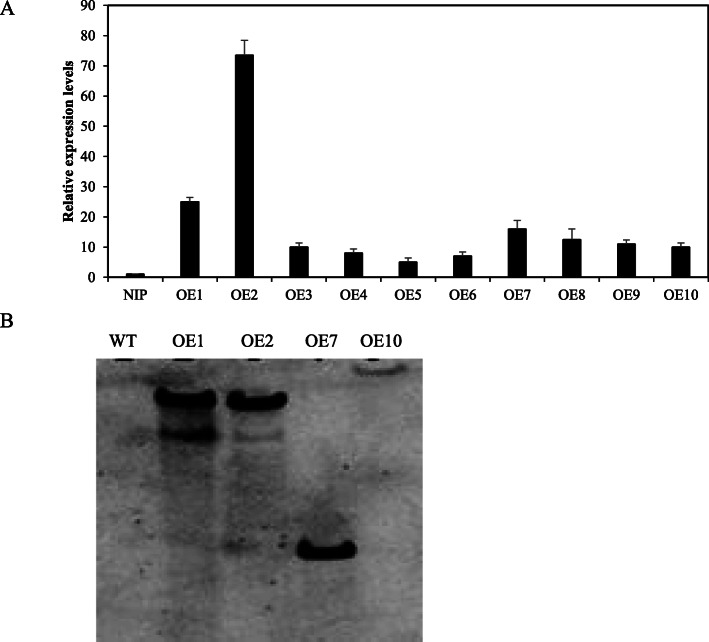
Identification of transgenic lines overexpressing *OsSTAP1.***a***OsSTAP1* expression analysis in the transgenic lines. Error bars showing the SD are based on three replicates. *Actin* was used as the endogenous control for normalization of gene expression. **b** Southern blotting detection of *OsSTAP1* in the transgenic rice lines. WT (wild type) as the negative control

*OsSTAP1* is induced by multiple abiotic stress and hormonal treatments, suggesting that *OsSTAP1* may play an important role in hormone signaling and the stress response. To examine the regulatory function of *OsSTAP1*, we constructed transgenic lines overexpressing *OsSTAP1*. The expression levels of *OsSTAP1* in the OE lines, especially in OE1, OE2, and OE7, were increased considerably compared to WT ‘Nipponbare’ as determined by qRT-PCR analysis (Fig. 4a). Four independent transgenic lines, OE1, OE2, OE7, and OE10, were analyzed by Southern blotting to quantify the number of transgene insertions, and two transgenic lines, OE1 and OE2, with two insertions each were then selected for further characterization of the salt-tolerant phenotype (Fig. 4b).

**Fig. 5:**
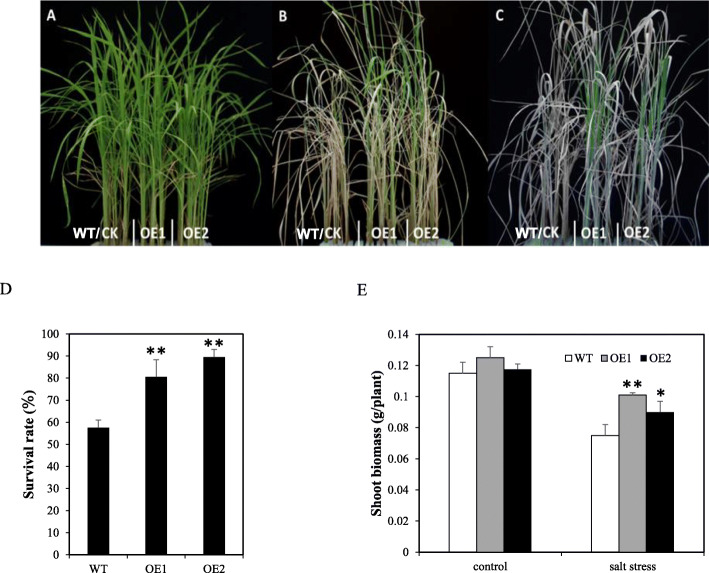
Salt stress in the *OsSTAP1*-overexpression lines. Phenotypes of WT and *OsSTAP1* OE plants at the seedling stage in response to salt stress: before treatment (**a**), 150 mM NaCl for 5 days (**b**), and recovery for 7 days (**c**). Survival rates after recovery for 3 days (**d**), and shoot biomassof the OE and WT plants under the control and salt stress conditions (**e**). Each column are means ±SD (*n* = 3. * and ** indicate significant difference at *P*<0.05 and *P*<0.01, respectively, based on Dunnett’s multiple comparison tests in ANOVA)

Further phenotypic observation showed that the morphological traits of the two transgenic lines at seedling stage were not significantly different from those of the wild type under normal conditions (Fig. 5a). While, OE1 and OE2 lines suffered less damage and were less sensitive to salt stress compared to WT after salt treatment (150 mM) for 5 days (Fig. 5b). After recovery for 7 d, the survival rates were counted, ~ 62% in WT and 81–91% in the OE lines (Fig. 5c). Compared to WT, the OE lines exhibited improved salt tolerance with significantly higher seedling survival rates after recovery for 3 d (Fig. 5d). Besides, OE lines had higher shoot biomass than WT under salt stress condition, while no significant differences between the WT and OE lines were found under normal condition (Fig. 5e). These results show that *OsSTAP1* acts a positive factor in response to salt stress in rice seedlings.

### Physiological and Biochemical Changes in the Lines Overexpressing *OsSTAP1*

**Fig. 6:**
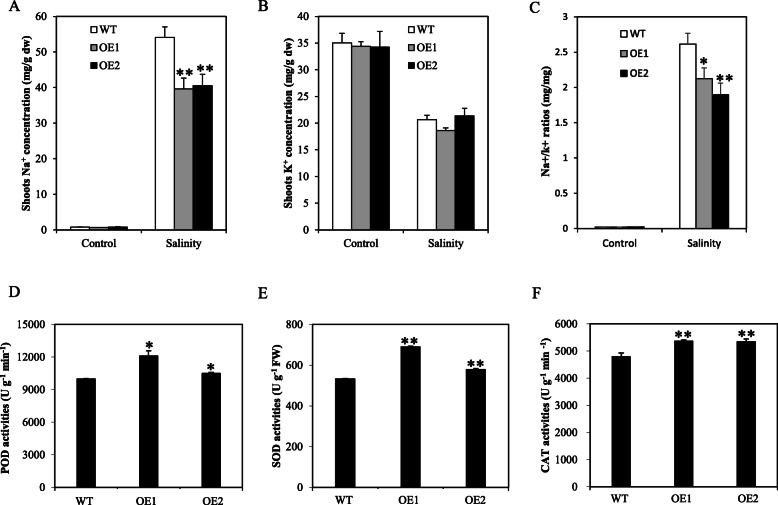
Physiological trait performances in WT and *OsSTAP1*-OE plants in response to salt stress. Determination of Na^+^ (**a**) and K^+^ (**b**) concentrations, the Na^+^/K^+^ ratios (**c**) and the activities of POD (**d**), SOD (**e**), and CAT (**f**) in rice shoots under salt stress. Each column are means ±SD (*n* = 3. * and ** indicate significant difference at *P*<0.05 and *P*<0.01, respectively, based on Dunnett’s multiple comparison tests in ANOVA)

Salt tolerance in plants is the consequence of several physiological processes, such Na^+^ uptake, Na^+^/K^+^ balance, and ion compartmentalization. Damage to leaves caused by salt stress is attributed to accumulation of Na^+^ in the shoots by transport of Na^+^ from the roots when exposed to high external salt concentrations (Lin et al. 2004; Liu et al. 2012b). Thus, in the current study, we measured the accumulation of Na^+^ and K^+^ in the rice shoots. There was no significant difference in K^+^ content between WT and OE lines before or after salt treatment. The accumulation of Na^+^ in OE line was dramatically lower than in the WT after salt treatment, although there was no difference in Na^+^ content between the WT and OE lines before salt treatment (Fig. 6a). The Na^+^/K^+^ ratios in the shoots were calculated based on the Na^+^ and K^+^ contents, which markedly decreased (Fig. 6c). These results support that overexpression of *OsSTAP1* could significantly reduce the content of sodium ion and the ratio of Na^+^/K^+^ in the shoots of plants exposed to salt stress.

Furthermore, to investigate physiological and biochemical variation in salt tolerance of the OE lines, we measured the activities of antioxidant enzymes such as CAT, POD, and SOD. Two OE lines both showed remarkably higher activities of antioxidant enzymes (Fig. 6d-e). Above results concluded that overexpression of *OsSTAP1* can significantly induce the activities of antioxidant enzymes in rice plants under salt stress, which may enhance the scavenging of reactive oxygen species (ROS).

### Overexpression of *OsSTAP1* Was less Sensitive to ABA

**Fig. 7:**
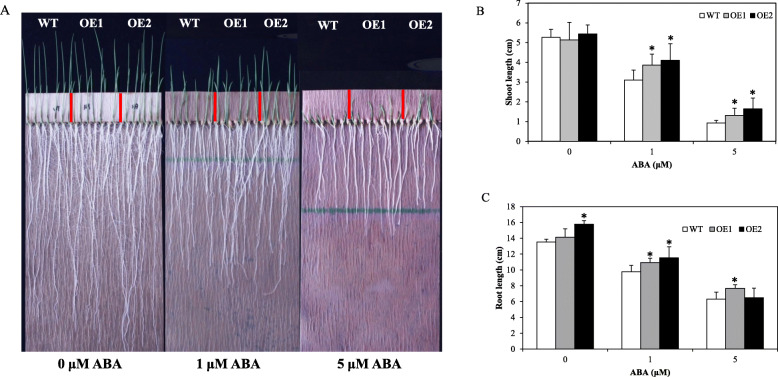
Sensitivity of the OE plants to ABA. The phenotypes (**a**), shoot lengths (**b**) and root lengths (**c**) of WT and OE lines grown in germination bags under different concentrations of ABA. Each column are means ±SD (*n* = 20. * indicates significant difference at *P*<0.05 based on Dunnett’s multiple comparison tests in ANOVA)

ABA is a phytohormone that plays an important role in response to abiotic stress. Our previous results showed that ABA could up-regulate the expression of *OsSTAP1* (Jin et al. 2009). Plants overexpressing *OsSTAP1* were less sensitive to ABA (Fig. 7a). The shoots length of both OE lines and roots length of OE1 line were much longer than WT under 1 μM and 5 μM ABA treatments. For the OE2 line, the roots length was significantly longer than the WT under 1 μM ABA and non-ABA conditions, but there was no significant difference between WT and OE2 under 5 μM ABA (Fig. 7b, c). Therefore, *OsSTAP1* may participate in the regulation of growth and development by ABA, and overexpression decreased the suppression of ABA to plant growth.

### Downstream Genes and Pathways Involved in Salt Stress Regulated by *OsSTAP1*

To gain insights into the genes regulated by *OsSTAP1*, we performed transcriptome sequencing of OE1 and WT plants under normal growth conditions and salt stress. One hundred four and twenty-four genes were up- and down-regulated in OE1, respectively, compared to WT under normal growth conditions (Supplementary Table S2). Among the genes up-regulated in the OE line, *Os02g0745100* encodes an Aquaporin NIP2–1 protein that is responsible for silicon transport from the external solution to the root cells (Ma et al. 2006). Silicon promotes plant growth and participates in response to biotic and abiotic stresses by preventing lodging (falling over) (Ma et al. 2006). *Os02g0658100* encodes an aquaporin, which can facilitate the transport of water and small neutral solutes across cell membranes (Shekoofa and Sinclair 2018). It is worth noting that two ethylene-responsive transcription factor genes, *ERF113* (*Os04g0398000*) and *ERF1* (*Os03g0183000*), were up-regulated in OE1. It has been reported that ERF113 is involved in the regulation of plant development and acts as a positive regulator of tolerance to abiotic stress by regulating antioxidant enzyme activities through the ABI1-mediated ABA signaling pathway in *Arabidopsis thaliana* (Krishnaswamy et al. 2011; Liu et al. 2012a). In addition, two peroxidase genes, encoding peroxidase 72 (*Os04g0423800*) and peroxidase 1 (*Os07g0104500*), were also up-regulated in OE1 plants. Peroxidases can scavenge intracellular hydrogen peroxide and reduce the damage caused by ROS, maintaining the redox balance in plants (Novo-Uzal et al. 2014). All of the genes described above may mediate the higher salt tolerance observed in the OE1 plants.

Under salt stress, there were 43 and 16 genes that were up- and down- regulated, respectively, in OE1 as compared to WT (Supplementary Table S3). Similar to normal growth conditions, *Os03g0183000* (*ERF1*) was also induced in OE1 under salt stress, which suggests that *ERF1* may be the one of the candidate targets of OsSTAP1 and play an important role in response to salt stress. *Os12g0169300* that encodes glutathione S-transferase T3, was also up-regulated in the OE line. Glutathione S-transferases (GSTs) are a well-characterized detoxification enzyme family involved in stress tolerance, which catalyzes the conjugation of reduced tripeptide glutathione (GSH) to electrophilic substrates (Sharma et al. 2014). Compared with the WT, the expression level of *Os03g0576200* (*OsHAK21*) and *Os01g0307500* (*OsHKT8*) increased in the OE line under salt stress. *OsHAK21* encodes a member of high-affinity K^+^ transporter. *oshak21* was found sensitive to salt stress, accumulated less K^+^ and considerably more Na^+^ in both shoots and roots, and had a significantly lower K^+^ net uptake rate but higher Na^+^ uptake rate (Shen et al. 2015). *Os01g0307500* encodes a sodium transporter (HKT8), and HKT8 protein is a specific transport protein of sodium ion (Hauser and Horie 2010). Under salt stress, *OsHKT8* returns excessive Na^+^ from shoot to root through the xylem, so as to reduce the toxicity of Na^+^ and enhance the salt tolerance of rice (Ren et al. 2005). To further identify the target genes of *OsSTAP1*, the promoter regions (2000 bp upstream of the transcription start site) of the 43 genes up-regulated in the OE line were analyzed, and the results showed that there were many binding sites for ERF TFs, GCC box, and DRE/CRT *cis*-elements (Supplementary Table S1), indicating that these genes are most likely directly regulated by *OsSTAP1* and function in response to salt stress in rice.

To confirm the accuracy and reliability of the transcriptome data, we detected the expression level of 24 genes by qRT-PCR, including the major genes described in results such as *ERF113*, *ERF1*, aquaporin NIP2–1, peroxidase 72, and peroxidase 1. The results showed that the expression level by transcriptome was highly correlated with that detected by qRT-PCR with R^2^ = 0.9739 (Figure S1, Supplementary Table S5).

## Discussion

This study reports the characterization of *OsSTAP1*, which was classified into phylogenetic group ERF-type superfamily proteins by Nakano et al. (2006). Genes in this group have been shown to play crucial roles in biotic and abiotic stress responses. A wheat ERF gene, *TaERF1*, which was induced by multiple environmental stresses and exogenous hormones such as drought, salt, ABA, ET, and salicylic acid (SA), was identified as a stress-related gene that could enhance tolerance to multiple abiotic stresses (Xu et al. 2007). A salt-inducible *ERF*-type gene, *ERF38*, improved salt and osmotic tolerance by regulating the expression of several POD and SOD-related genes in transgenic poplar lines (Cheng et al. 2019). Additionally, *GmERF75* could enhance osmotic tolerance in both *Arabidopsis* and soybean by increasing the chlorophyll content under drought and salt stress conditions. Overexpressing *GmERF75* in soybean hairy roots improved root growth in response to exogenous ABA and salt stress (Zhao et al. 2019). In our study, we found that *OsSTAP1* was induced by multiple abiotic stress, including drought, salt, and low temperature, and there were many CRT/DRE cis-elements present in the promoter region. Genes encoding aquaporins, peroxidases, and other ERF TFs were up-regulated in OE1 plants, indicating that they could be the target genes of OsSTAP1. We speculated that *OsSTAP1* not only played a role in response to salt stress, but also probably taked part in osmotic regulation, drought, and other stresses via regulating different downstream target genes.

ERF transcription factors are targets of different signaling pathways (Xu et al. 2008). For example, ERF1 integrated signals from the ethylene and jasmonate pathways and mediated resistance to the soilborne fungus *Fusarium oxysporum* in *Arabidopsis* (Berrocal-Lobo and Molina 2004; Lorenzo et al. 2003). ERF4 acted as a transcriptional repressor modulating abscisic acid and ethylene responses (Yang et al. 2005). *OsDRAP1*, which encodes a DREB2-like TF, affected drought tolerance by redox homeostasis and maintaining water balance in rice in an ABA-dependent manner (Huang et al. 2018). *OsSTAP1* expression was induced by multiple phytohormones, including ABA, ACC, and the promoter contained many ABREs. It was worth noting that the OE lines were less sensitive to the ABA suppression to plant growth. All of the results suggest that *OsSTAP1* enhances the tolerance to salt stress, which is mediated by the ABA-dependent signaling pathway.

Ion toxicity (mainly Na^+^) to cells is a primary stress signal caused by salt stress, and plants use a calcium-dependent protein kinase pathway (SOS) to maintain a high K^+^/Na^+^ ratio in the cytoplasm and to mediate salt stress signaling (Zhu 2002; Zhu 2016). The secondary effects of salt stress are complex. Salt stress can alter the balance of reactive oxygen species (ROS) and lead to oxidative cellular damage, including membrane lipid destruction, protein and nucleic acid damage, and metabolic disorders. Plants can enhance their tolerance to salt stress by increasing the activities of antioxidant enzymes or the contents of antioxidants (Zhu 2002). In our study, there were higher K^+^/Na^+^ ratios, and the activities of antioxidant enzymes, including CAT, POD, and SOD, were increased in the OE lines compared to WT under salt stress. Additionally, many genes related to ROS scavenging, such as glutathione S-transferase T3, peroxidase 72 and Peroxidase 1, were induced by *OsSTAP1*. Salt tolerance in plants is a complex biological process; *OsSTAP1*, which acts as a transcriptional activator, induces the expression of different genes to decrease the Na^+^/K^+^ ratio and increase activity of antioxidant enzymes in the shoots, ultimately increasing the tolerance to salt stress. However, the detailed molecular mechanisms and genetic pathways of *OsSTAP1*-mediated salt tolerance remain to be elucidated.

## Conclusion

*OsSTAP1,* an ERF-type transcription factor, enhances salt stress tolerance by decreasing Na^+^/K^+^ ratios and increasing the activities of antioxidant enzymes in the shoots. Moreover the expression of *OsSTAP1* is induced rapidly by multiple abiotic stress and phytohormone treatments, and plants overexpressing *OsSTAP1* are less sensitive to the ABA-mediated suppression to plant growth and development. OsSTAP1 is a transcriptional activator localized to the cell nucleus that can induce the expression of many stress-related genes such as *ERF113*, *ERF1*, aquaporin NIP2–1, peroxidase 72, and peroxidase 1 in response to salt stress. All of the results in our study suggest that *OsSTAP1* is a potential candidate gene for the improvement of salt tolerance in rice.

## Materials and Methods

### Plant Growth Conditions and Stress Treatments

To detect the transcription level of *OsSTAP1* under various abiotic stresses and phytohormone treatments, seeds of *Oryza sativa ssp. japonica* cv. ‘Nipponbare’ were sterilized in 0.1% NaClO (v/v) for 24 h and placed in an incubator at 37 °C for ~ 48 h until germination. The rice plants were watered with Yoshida solution in the greenhouse for 3 weeks as described by Wang et al. (2011). Seedlings at the four-leaf stage were exposed to different stress treatments including low temperature (4 °C), high salinity (150 mM NaCl), high osmotic pressure with 20% (w/v) PEG, and hormone treatments consisting of 100 μM ABA, 100 μM ACC, or 100 μM 6-BA. Shoot samples were collected at 0, 1, 3, 6, 9, 12, and 24 h and frozen immediately in liquid nitrogen.

### RNA Isolation and qRT-PCR Gene Expression Analysis

Total RNA was extracted from frozen tissues using TRIZOL reagent (Invitrogen, USA) and then purified and concentrated with the RNeasy MinElute Cleanup kit (Qiagen, Germany; catalog no. 74204). Quantitative real-time PCR (qRT-PCR) was performed with SYBR® Premix Ex Taq™ (TaKaRa, Japan). The rice *Actin* gene was used as the endogenous control for data normalization and the 2^-∆∆Ct^ method was used to calculate the relative expression of each gene. Each assay was repeated three times as described previously (Wang et al. 2011). The nucleotide sequences of the primers used were given in Supplementary Table S4.

### Sequence Analysis

Genes homologous to *OsSTAP1* were identified by BLAST searches of the NCBI database (http://www.ncbi.nlm.nih.gov/BLAST/). A phylogenetic tree was constructed using the neighbor-joining method as implemented in MEGA5 software. Numbers at the nodes indicated bootstrap percentage values after resampling the data 1000 times. The multiple sequence alignment was performed with ClustalW. We searched the promoter sequence (1500 bp upstream from the transcription start site) against the PLACE database (https://www.dna.affrc.go.jp/PLACE/?action=newplace) to identify possible DNA motifs present in cis-acting elements that may reveal potential regulation of *OsSTAP1*.

### Plasmid Construction and Plant Transformation

The full-length CDS of *OsSTAP1* was amplified from ‘Nipponbare’ using gene-specific primers (*OsSTAP1*-PB) that incorporated *Pst*I and *Bam*HI restriction sites at the 5′ and 3′ ends. After digestion with the two restriction endonucleases, the *OsSTAP1*gene was cloned into the pCUbi1390 vector under control of the ubiquitin 1 promoter. The vector was transformed into *Agrobacterium tumefaciens* strain EHA105 and the resulting strain was used for rice transformation following the protocol of Duan et al. (2012).

### Subcellular Localization of OsSTAP1

The complete *OsSTAP1* CDS without the stop codon was amplified using the primer pair *OsSTAP1*-EA and cloned into the pGWC vector. *OsSTAP1* was transferred into the PMDC43 vector by homologous recombination using Gateway™ cloning technology to produce the OsSTAP1-GFP fusion construct. The recombinant construct and free GFP were introduced separately into tobacco (*Nicotiana tabacum* L.) leaf epidermal cells with a model PDS-1000/He Biolistic particle delivery system (Bio-Rad, CO, USA). After incubation at 25 °C for 72 h, the green fluorescence signal was observed using a confocal laser scanning microscope (LSM510 Meta, Carl Zeiss; http://www.zeiss.com/) with an argon laser excitation wavelength of 488 nm.

### Transactivation Analysis in Yeast

Transactivation of OsSTAP1, an ERF-type transcription factor, was examined in a yeast assay system as described by Liu et al. (2013). The CDS fragment of *OsSTAP1* that was amplified by PCR using *Eco*RI and *Bam*HI linker primers (*OsSTAP1*-EB) was double digested and ligated with the vector pGBKT7-BD generating BD-OsSTAP1. BD-OsSTAP1 and pGBKT7 were transformed into yeast strain *AH109* in the Matchmaker Gold Yeast Two-Hybrid System (Clontech, Japan) as directed by the manufacturer. Transcriptional activation activity of OsSTAP1 was evaluated based on the growth status of yeast on SD/−Trp and SD/−Trp-His-Ade media.

### Phenotypic and Physiological Characterizations

Rice seedings at the four-leaf stage were treated with 150 mM NaCl for 5 d and cultured in Yoshida solution without extra NaCl for 7 d. Phenotypes, including shoot and root lengths, dry weights (DW), and survival rates were measured for *OsSTAP1* OE lines and the wild type (WT) under salt stress as well as control conditions. The root and shoot samples were weighed after drying at 70-75 °C for 72 h, and seedling survival rates were calculated as the ratio of surviving plants over the total number of treated plants. Seedings with four leaves were also treated with 120 mM NaCl for 24 h, and sodium and potassium concentrations and antioxidant enzyme activity were assayed according to the method described by Zang et al. (2008) and Bonnecarrère et al. (2011). To detect the Sensitivity of the OE lines to ABA, seeds of the WT and OE lines were sterilized in 0.1% NaClO (v/v) for 24 h and placed in an incubator at 37 °C for ~ 48 h until germination. The germinated seeds were placed in germination bags and watered with 0 μM, 1 μM, and 5 μM ABA. The root length and shoot length under different treatments were observed and recorded.

### Transcriptome Analysis

The *OsSTAP1* overexpressing line (OE1) and WT (‘Nipponbare’) were grown in the greenhouse and watered with Yoshida solution as described above. Seedlings at the four-leaf stage treated with 150 mM NaCl for 4 d and the controls (no NaCl) were collected and frozen immediately in liquid nitrogen. There were three replicates of each treatment, with four seedlings per replicate. Transcriptome sequencing was performed by Annoroad Gene Technology (Beijing, China) on an Illumina sequencing platform. The integrity and concentration of RNA samples were determined with an Agilent 2100 RNA nano 6000 assay kit (Agilent Technologies, CA, USA). Differentially expressed genes (DEGs) were identified using the |log2ratio| 1 and Q < 0.05 criteria (Wang et al. 2010).

## Supplementary information

**Additional file 1: Table S1.** Cis-elements analysis of the *OsSTAP1* promoter sequence. **Table S2.** List of the differentially expressed gene in the *OsSTAP1* overexpression line compared with WT under normal growth conditions. **Table S3.** List of the differentially expressed gene in the OsSTAP1 overexpression line compared with WT under salt stress conditions. **Table S4.** The primers used in the present study. **Table S5.** The expression levels of 24 differentially expressed gene detected by transcriptome and qRT-PCR.

**Additional file 2: Figure S1.** The correlation analysis of the expression levels of 24 DEGs detected by transcriptome and qRT-PCR.

## Data Availability

Not applicable.

## Abbreviations

6-BA: N-(Phenylmethyl)-9H-purin-6-amine
ABA: Abscisic acid
ABRE: Abscisic acid response element
ACC: 1-aminocyclopropane-1-carboxylate
AP2/EREBP: APETALA2/ethylene-responsive element binding protein
AP2/ERF: APETALA2/ethylene responsive factor
ARFAT: Auxin response factor binding site
bHLH: Base helix-loop-helix transcription factors
bZIP: Basic leucine zipper transcription factors
CAT: Catalase
CDS: Coding sequence
DW: Dry weights
GA: Gibberellin
GARE: Gibberellin response element
GFP: Green fluorescent protein
LTR: Low temperature response element
MYB: V-myb avian myeloblastosis viral oncogene homolog transcription factors
NAC: NAM、ATAF1/2、CUC2 transcription factors
OE: Over-expressing
PCR: Polymerase chain reaction
PEG: Polyethylene glycol
POD: Peroxidase
SOD: Superoxide dismutase
TFs: Transcription factors
WT: Wild type
