# Supplementary figures and images for: Overexpression of the Transcription Factor Gene OsSTAP1 Increases Salt Tolerance in Rice
Source: Rice (N Y). 2020 Jul 23;13:50. doi: 10.1186/s12284-020-00405-4 (PMC7378131; doi:10.1186/s12284-020-00405-4)

## Slide 1
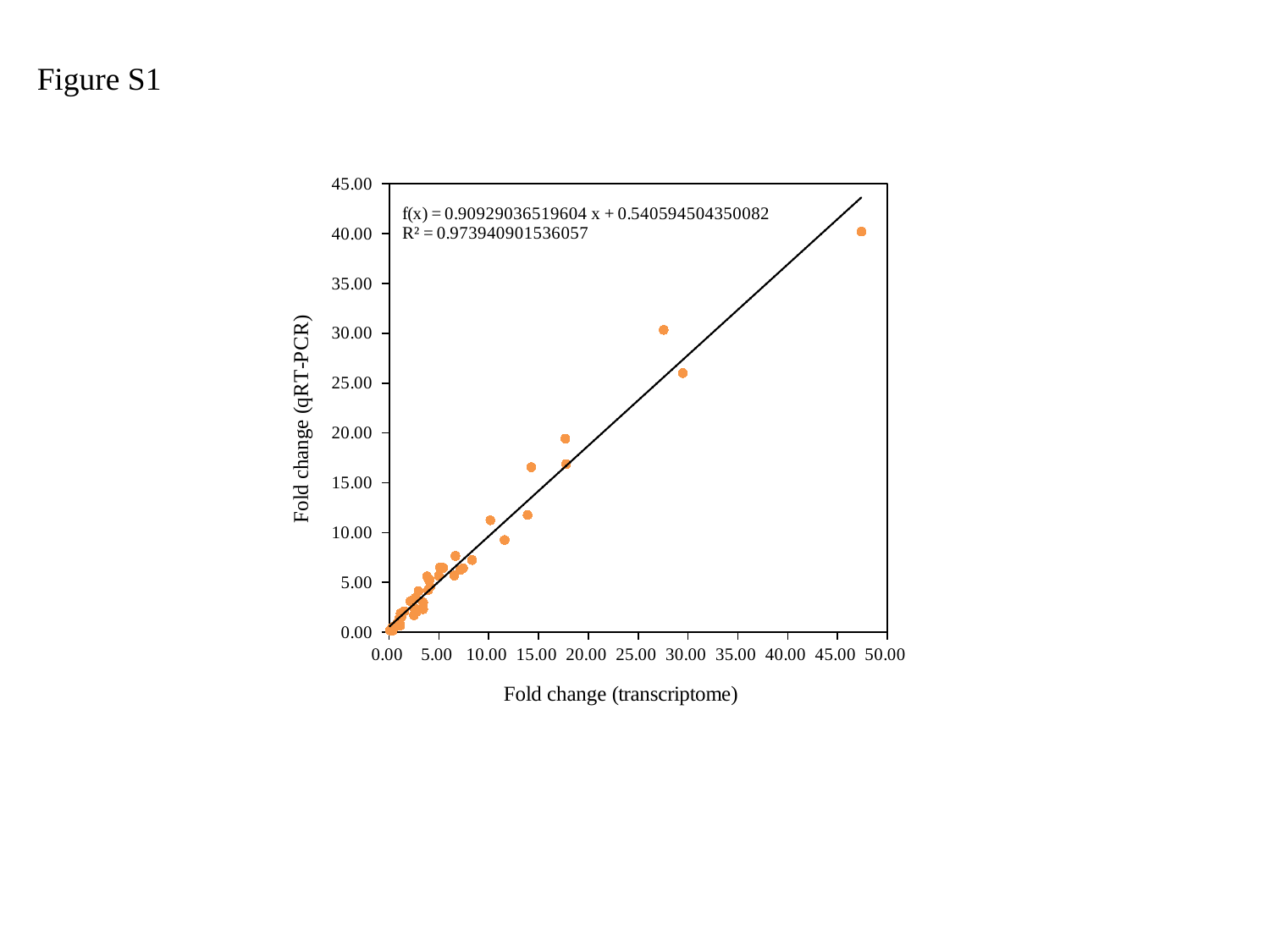

Figure S1
### Chart
| Category | |
|---|---|

Supplement: Supplementary file 2 — Additional file 2: Figure S1. The correlation analysis of the expression levels of 24 DEGs detected by transcriptome and qRT-PCR. [file 12284_2020_405_MOESM2_ESM.pptx]
